# Taking stock of Myanmar’s progress toward the health-related Millennium Development Goals: current roadblocks, paths ahead

**DOI:** 10.1186/1475-9276-12-78

**Published:** 2013-09-11

**Authors:** Yu Mon Saw, Khine Lae Win, Laura Wen-Shuan Shiao, Moe Moe Thandar, Rachel M Amiya, Akira Shibanuma, Soe Tun, Masamine Jimba

**Affiliations:** 1 Department of Community and Global Health, Graduate School of Medicine, The University of Tokyo, 7-3-1 Hongo, Bunkyo-ku, Tokyo 113-0033, Japan; 2 Department of Preventive and Social Medicine, The University of Medicine 2, North Okkalapa Township, Yangon 11031, Myanmar

**Keywords:** Millennium Development Goals, Global health, International cooperation, Financial support, Developing countries, Myanmar

## Abstract

Myanmar is a developing country with considerable humanitarian needs, rendering its pursuit of the Millennium Development Goals (MDGs) an especially high priority. Yet progress to date remains under-examined on key fronts. Particularly within the three health-related MDGs (MDGs 4, 5, and 6), the limited data reported point to patchy levels of achievement. This study was undertaken to provide an overview and assessment of Myanmar’s progress toward the health-related MDGs, along with possible solutions for accelerating health-related development into 2015 and beyond. The review highlights off-track progress in the spheres of maternal and child health (MDGs 4 and 5). It also shows Myanmar’s achievements toward MDG 6 targets – in the areas of HIV/AIDS, malaria, and tuberculosis. Such achievements are especially notable in that Myanmar has been receiving the lowest level of official development assistance among all of the least developed countries in Asia. However, to make similar progress in MDGs 4 and 5, Myanmar needs increased investment and commitment in health. Toward moving forward with the post-2015 development agenda, Myanmar’s government also needs to take the lead in calling for attention from the World Health Organization and its global development partners to address the stagnation in health-related development progress within the country. In particular, Myanmar’s government should invest greater efforts into health system strengthening to pave the road to universal health coverage.

## Introduction

Myanmar is in a phase of historic transition, both in the domestic sphere and in its external relations. Once tipped by the World Bank as destined for prosperity [1], the country has yet to see this promise realized – to the detriment of its economic and social development. The population in 2009–2010 was sixty million, with roughly 70% living in rural areas and engaged in agriculture [2]. The country is replete with resources, yet still ranks consistently as a low-income country and one of the least developed countries in the Asia-Pacific. Meanwhile, the disparities between urban and rural development levels are increasing, particularly in the realms of employment opportunities, access to health care services, and education [2]. The country’s Human Development Index was 0.483 in 2011, ranking in at 149 out of 187 countries globally [3].

Now, after decades of military rule, political reforms are finally in motion and Myanmar is opening itself up to increasing engagement with the outside world. The international community has responded by starting to lift sanctions, and the return of development assistance is likewise expected. It is a moment of opportunity for renewed commitment to development and health – areas that have seen slow progress for decades.

As one of the 189 signatories to the United Nations Millennium Declaration of 2000, Myanmar decided to work toward achieving the eight Millennium Development Goals (MDGs) by 2015. Although only two years remain until that cut-off date, performance in achieving the health-related MDGs has not been uniform across the programs in Myanmar. Early gains have brought the country on track for targets to halve prevalence of HIV, tuberculosis (TB) and malaria. However, reducing child and maternal mortality toward MDGs 4 and 5 remain major challenges [4].

This review provides an overview of current trends in Myanmar’s pursuit of the health-related development goals and aims to identify bottlenecks to progress. By examining the issues at the crux of the hold-up in the Myanmar context, we propose possible solutions for accelerating positive health-related achievements into 2015 and beyond.

### Myanmar’s progress on the health-related MDGs

#### MDGs 4 & 5: Falling short on maternal and child health

Myanmar has shown some progress over the past two decades in reducing child mortality toward MDG 4 targets. The under-five mortality rate (U5MR) is trending downwards, falling from 115 per 1,000 live births in 1990 to 80 in 2000 and 54 in 2010 (Table 1) [5]. However, extrapolating from the current trajectory, Myanmar is unlikely to reach the overall U5MR target of 38 or fewer per 1,000 live births by 2015. The infant mortality rate (IMR), similarly, has also fallen in the past 20 years, from 77 per 1,000 live births in 1990 to 62 in 2000 and 49 in 2010 (Table 1). Still, Myanmar remains off-track to fulfill the MDG IMR target of 26 or fewer per 1,000 live births by 2015 [5]. However, measles immunization coverage has increased from 68% in 1990 to 88% in 2010, and Myanmar is expected to meet the 2015 target of 90% or greater coverage [5].

**Table 1:** Trends in health-related MDG indicators in Myanmar, 1990-2010

**MDG indicator**		**1990**	**1995**	**2000**	**2005**	**2010**	**Actual % reduction achieved**	**Goal for 2015**
**MDG 4: Reduce by two-thirds, between 1990 and 2015, the under-5 mortality rate.**	*4.1 ****Under-5 mortality rate***^***a***^	115	91	80	61	54	53.0	38
	*4.2 ****Infant mortality rate***^***a***^	77	69	62	55	49	36.0	26
**MDG 5: Reduce by three quarters, between 1990 and 2015, the maternal mortality ratio.**	*5.1 ****Maternal mortality ratio (per 100,000 live births)***^***a***^	520	380	300	230	200	62.0	130
**MDG 6: Combat HIV/AIDS, malaria, and other diseases.**	*6.1 ****HIV prevalence among those aged 15–24 years***^a*^	0.2	0.6	0.8	0.7	-	-	-
	*6.6 ****Malaria incidence and malaria-associated death rates (malaria morbidity)***^***b***^	24.4	14.6	11.8	9.5	11.7	52.0	12.2
	*6.6 ****Malaria incidence and malaria-associated death rates (malaria mortality)***^***b***^	12.6	8.4	5.5	3.1	1.3	89.7	6.3
	*6.9 ****TB incidence***^***a***^	393	404	412	403	384	2.0	197
	*6.9 ****TB prevalence***^***a***^	894	881	831	647	525	41.0	447
	*6.9 ****TB-associated death rates among HIV-negative people***^***a***^	113	115	104	66	49	57.0	57

^a^Source: World Health Organization, Global Health Observatory.

^b^Source: Ministry of Health, Health in Myanmar 2012.

*Available data were incomplete for this metric.

Regarding improvements in maternal health toward MDG 5, meanwhile, the maternal mortality rate (MMR) has declined from 520 maternal deaths per 100,000 live births in 1990 to 200 in 2010. From 1990 to 2010, the MMR fell by 62%, at which rate the target of a three-quarters reduction for the period 1990–2015 can be achieved (Table 1) [6]. Progress, however, has been patchy along the way. Following significant 5-year-interval rates of decline from 1990, the improvements slowed down considerably between 2005 and 2010 (Table 1) [5]. Beyond the MMR, a slow upward trend in key maternal health indicators, including skilled birth attendance, antenatal care coverage and contraceptive uptake, was observed between 1990 and 2010 in Myanmar, though information on some indicators is unavailable [7,8].

The stagnation in progress observed between 2005 and 2010 was likely due to dynamics encompassing natural disaster, the limited resources available, and socioeconomic factors. For instance, Myanmar was severely affected in 2008 by Cyclone Nargis, the worst natural disaster in Myanmar’s recorded history. As a result, some US$ 10 billion in damages were incurred and over 133,000 people died [9]. Regarding available resources, the Global Fund unexpectedly withdrew from Myanmar in 2005 [10], which had major ramifications for health and development efforts. The resulting loss of funds might have contributed to the observed MMR trend. Furthermore, the out-of-pocket (OOP) expenditure on health has increased from US$ 4 in 1995 to US$ 15 in 2010 [11]. This indicates that catastrophic financial costs became a major barrier for poor of lower socioeconomic groups women to access health services.

### Progress on MDG 6: Relatively on-track against the “big three” diseases

Myanmar has made major strides in combatting the “big three” infectious diseases addressed in MDG 6 (HIV/AIDS, malaria, and TB). The country’s HIV prevalence among adults aged 15 to 49 years peaked at 0.8% in 2000, but rates fell to 0.7% in 2005 (Table 1) [5]. At the intersection with TB, progress is, unfortunately, less consistent; HIV prevalence among newly diagnosed TB patients has fluctuated between 1% and 10% since 2005 [2]. Still, among the most-at-risk groups – including men who have sex with men, female sex workers and injecting drug users – HIV prevalence rates have significantly declined [2].

Malaria morbidity, meanwhile, fell from 24.4 to 11.7 per 1000 population in the period from 1990 to 2010, while mortality declined from 12.6 to 1.3 per 100,000 population in Myanmar [2]. Appreciable progress has been made, too, in TB control, as measured in both case detection and treatment success rates. TB case detection rate increased from 8% in 1990 to 71% in 2010, and TB treatment success rates rose from 77% in 1994 to 85% in 2009 [5]. Deaths due to TB among HIV-negative people have also been reduced from 110 per 100,000 population in 1990 to 41 per 100,000 in 2010 (Table 1) [5].

Advances made in reaching MDG 6 goals in Myanmar have largely been a product of effective disease-specific vertical programs [2]. For instance, through a host of such programs, Myanmar has successfully promoted the use of insecticide-treated nets (ITNs), rapid diagnostic test and artemisinin-based combination therapy to reduce malaria-related morbidity and mortality. These disease-specific programs have been implemented through close collaboration with the local and international non-government organizations (NGOs), donors, and UN agencies [2].

### Major health-related development roadblocks to be overcome in Myanmar

Clearly, Myanmar has achieved notable headway in pursuing certain facets of the health-related MDGs. At the same time, the country faces critical challenges arising from a combination of internal and external operating environment factors that constrain public health program implementation and scaling up.

#### Insufficient, inconsistent investment in public health

Adequate and certain resource flows are critical for program development and service delivery – and sorely lacking in the context of Myanmar’s public health response toward achieving the health-related MDGs. Government expenditure on health was increased three-fold between 2000–2001 and 2005–2006 in Myanmar, but total expenditure on health as a percentage of gross domestic product (GDP) was stagnant at around 2% across these years [12]. Notably, Myanmar has among the lowest government expenditures on health in the region: US$ 4 per capita in 2010. In the 2011–2012 fiscal year, the government increased its expenditure on health to a level four times higher than in previous years. However, OOP payments in Myanmar are among the highest globally, going from 99% in 2005 to 92% in 2010 [12], constituting almost all of health expenditures in Myanmar. In this way, the system of health financing in Myanmar only exacerbates existing health inequalities, interfering with development efforts.

**Figure 1:**
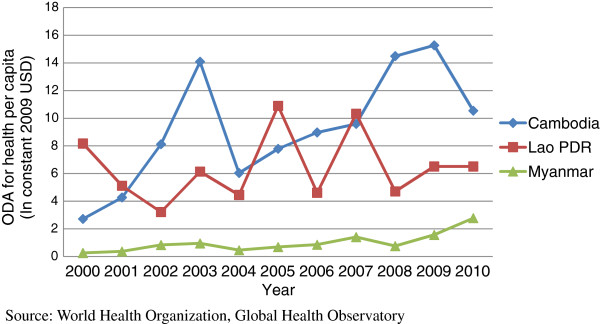
Official development assistance for health per Capita 2000-2010.

Myanmar receives a very low level of international financial assistance considering its development profile. Compared against other nearby Southeast Asian countries, Myanmar received the lowest level of official development assistance (ODA; US$ 338.84 million) in 2010, and ODA for health per capita was still the lowest compared to Cambodia and Lao PDR (already lower-middle income countries) (Figure 1) [12]. Moreover, the ODA expended for health-related purposes in Myanmar went mainly toward MDG 6-related programs (67.5%), with only 8.6% going toward reproductive health and family planning, in 2009 [13]. Such funding shortfalls are clearly reflected in Myanmar’s lagging health outputs.

#### Compromised service delivery and lack of health workforce

Myanmar’s public health care system is critically under-resourced, with major gaps in access and coverage. The health system is structured in line with the country’s administrative structure, with health departments at state/regional, district and township levels [14]. For example, the township-level health facilities encompass the township hospital, station hospitals, and rural and sub-rural health centers [15]. A lack of human resources in peripheral regions and a paucity of drugs and of basic information for monitoring are critical roadblocks to extending public health service coverage [16]. Moreover, the country is plagued by severe inequalities in infrastructure, program coverage and transportation access. Furthermore, at the township level, an overemphasis on vertical programming weakens the health system. Township-level health staff, meanwhile, are not equipped organizationally or managerially to meet the challenges of maternal and child health care [17-19].

Myanmar is one of 57 countries facing a severe health workforce crisis as designated by WHO, with only 1.3 health workers (doctor, nurse, and midwife) per 1000 population [20]. In the South and Southeast Asia region, insufficient skilled birth attendants represents perhaps the biggest challenge for combatting maternal mortality [21]. As with other countries in the region, Myanmar’s ability to reduce its MMR depends heavily on such skilled birth attendants, especially midwives in emergency obstetric care and neonatal resuscitation [22]. Though the MOH has set a national target of at least one midwife in each village [23], the reality continues to lag behind the ideal, and scale-up of midwifery training is still urgently needed in Myanmar. The current ratio of midwifery attendants, including auxiliary midwives, to a village is 1:2, falling far short of the MDG 5 target [24]. Gaps in coverage are also evident with regard to malaria prevention; the proportion of children aged less than 5 years sleeping under long-lasting insecticide-treated nets (LLINs), for example, remains stubbornly low at around 11% [25].

The situation becomes even more dire in the country’s underserved regions. In Myanmar, 70% of the total population resides in rural areas, where resources and infrastructure are especially poor [2,26]. In many rural areas, health systems must contend with such hardships as poor electricity, limited transportation to reach health facilities, and absence of skilled health personnel.

#### Lack of statistical infrastructure

Fundamentally, Myanmar’s health system suffers from a lack of infrastructure along with a weak health information system for a variety of health indicators [6]. While the “big three” disease control programs have registered success stories, they still face important challenges [16]. Importantly, disease control programs are limited by reporting delays and weakness in analysis of disease surveillance information and response. A lack of quality data that is timely, complete and relevant is also evident in relation to the development of health information systems [18].

### On the cusp of 2015: recommendations for the final stretch and beyond

Current trends in Myanmar point to sizeable roadblocks still barring the country’s path to achieving all health-related MDGs. Though maternal and child mortality rates declined dramatically between 1990 and 2010, there is little chance of meeting MDG 4 and 5 targets by 2015. Regarding MDG 6, major strides forward in the programmatic response to the “big three” diseases notwithstanding, the country still faces critical challenges including bottlenecks to universal antiretroviral therapy (ART) coverage, resistant malaria parasites, and the emergence of new, multi-drug-resistant TB strains. Moving forward, Myanmar must address a number of critical roadblocks to progress while building on the momentum of its successes.

#### Sustaining and expanding upon successful disease control programs

As already mentioned above promising progress toward achievement of MDG 6 in Myanmar may be attributed largely to the relative success of disease-specific control programs, both public and private. For example, investments in HIV bring significant results in Myanmar, as in other countries in the region. At the same time, the lack of an appropriate, long-term, sustainable funding strategy represents a critical threat to the funding situation moving forward. This is currently resulting in a sizeable financing gap in the country’s National Strategic Plan on HIV and AIDS, with no specific plan to cover the funding gap. The Joint United Nations Programme on HIV/AIDS (UNAIDS) estimates that US$ 40 million are needed to fill the funding gap every year between 2012–2014 in Myanmar [4]. Such issues will need to be addressed if progress toward MDG 6 is to be sustained up to and beyond 2015. In the medium term, continued external support will likely be needed to help the country reach its HIV commitments [4].

A consistent decline has been observed, too, in deaths due to TB among HIV-negative people and in prevalence of TB generally. This is primarily the result of wide application of the most effective intervention recognized in this field (DOTS). Still, the incidence of TB remains high, perhaps due to discrepancies in socio-economic status and corresponding gaps in access to critical health services. Efforts must be made to close such gaps in service coverage to sustain and build upon progress made in combatting TB in Myanmar.

With regard to malaria mortality and morbidity, consistent declines have similarly been achieved in Myanmar. The major initiatives contributing to such progress include increasing accessibility to quality diagnosis, appropriate treatment according to national malaria treatment guidelines, and scaling up the ITN distribution program. LLINs are also distributed to endemic areas, with a special focus on children aged less than 5 years and pregnant mothers. Continuing to scale up such efforts in Myanmar will thus contribute to greater long-term progress in reducing the malaria burden.

#### International cooperation: Investing in public health

As in other resource-constrained countries, ODA in Myanmar plays an essential role to alleviate dire economic and humanitarian circumstances, and can also boost progress toward achieving the health-related MDGs [26]. With an increase in ODA for investment in health, Myanmar may yet have some prospect of getting on-track to achieve the health-related MDGs, particularly regarding maternal and child health [27]. While the international community has gradually lifted sanctions against Myanmar, donor states should also consider increasing funding in support of interventions targeting MDGs 4, 5, and 6.

Investments in HIV facilitate significant outcomes in Myanmar. While an HIV surveillance system was initiated in 1992, slow progress in the HIV/AIDS response had been observed due to the critical shortage of funding. Donors then established the Fund for HIV/AIDS in Myanmar in response to the HIV epidemic. By 2006, considerable advances had thus been made in the programmatic response toward service delivery [28]. However, the lack of an appropriate, long-term, sustainable funding strategy represents a critical threat to the funding situation moving forward. Such issues will need to be addressed if progress toward MDG 6, along with the other health-related MDGs, is to be sustained up to and beyond 2015. In the medium term, continued external support will likely be needed to help the country approach its MDG commitments [4].

#### Health systems strengthening

Myanmar has suffered chronically from infrastructure, program coverage, and transportation inequalities. A stronger health system and improved health workforce, both public and private, is critical to achieving the health-related MDGs. To this end, the country developed a health system strengthening (HSS) strategy and proposal through financial support from the Global Alliance for Vaccines and Immunization (GAVI) in 2007 and 2008. This HSS strategy was designed to close the gap in human resources management, program coordination and service delivery in order to reach the targets of MDGs 4 and 5 [17]. In 2011, assessments on various aspects of health systems at the township level were conducted, followed by financial management training programs in preparation for HSS implementation in 20 pilot townships. In December of the same year, the Coordinated Township Health Plan was endorsed to be conducted in the designated townships [2].

#### Moving from disease-specific programs to universal health coverage

Roadblocks notwithstanding, Myanmar has made considerable gains in utilizing its own resources to achieve MDG 6 targets. Modeling future strategies after the success of MDG 6 programming, a comprehensive maternal mortality reduction program may be mounted in Myanmar, in close coordination with existing child health programs. Moreover, underserved and vulnerable populations, such as those residing in rural areas, should be targeted specifically through maternal and child health initiatives [2,27]. To this end, Myanmar may consider introducing a universal health coverage scheme as a long-term investment [2,27]. Already Myanmar has initiated plans to introduce a Maternal and Child Health Voucher Scheme in one pilot township and a Township-Based Health Protection Scheme in another such pilot township [6]. Every country’s path to universal health coverage may be different; Myanmar’s government, for its part, should consider focusing on expansion of services to poor and vulnerable populations such as those residing in rural areas, elderly people, people living with disability, and those working in the informal sector.

#### Strengthening intersectoral cooperation for health

In Myanmar, almost all health services are delivered by the Ministry of Health (MOH). To tackle such public health work, the National Health Committee was formed 1989, through which most of the non-health ministries have been providing assistance for intersectoral cooperation [14]. For instance, the methadone maintenance therapy program was launched as a harm reduction project within HIV/AIDS control efforts, in collaboration with local and international NGOs, donors, and UN agencies. This has been carried out in collaboration with the MoH and the Ministry of Home Affairs [29]. Such positive steps in intersectoral cooperation must be built upon and expanded in working toward all of the health-related MDG goals.

In Myanmar, the situation in rural areas is particularly critical, with less coverage of skilled birth attendance, disadvantaged living conditions, and lack of transportation to reach health facilities [2,23]. To reduce child and maternal mortality, Myanmar needs to improve and strengthen intersectoral collaboration with non-health ministries such as the Ministry of Construction, the Ministry of Education, and the Ministry of National Planning and Economic Development.

### Toward a post-2015 development agenda

In Myanmar, disease-specific vertical programs such as those covering HIV/AIDS, malaria, and TB have been effective in bringing about achievements on the MDG 6 targets. Coordinated through the MOH, these initiatives have benefited from close collaboration with international and local NGOs. Based on such experiences, national programs for reduction of maternal and child mortality may also be initiated toward further progress on the health-related MDGs. At the same time, achievements made in Myanmar’s development must be sustained beyond 2015 and into the post-MDGs era. In particular, malaria control activities will continue to be critical even after 2015 because of the emergence of arteminisin-resistant parasites, although malaria mortality and morbidity have been impressively reduced over the last two decades. Building upon such achievements will thus be an important item to include on the next development agenda.

Beyond infectious diseases, the new development agenda must shift its focus more toward the emerging global burden of non-communicable diseases (NCDs), an issue notably absent from the current MDGs. Following global trends, Myanmar is now facing a double burden of diseases (both communicable and non-communicable) due to the demographic and socioeconomic transition occurring in the last several decades [2]. This is an issue that the post-2015 development framework must take into account in a comprehensive and meaningful way, incorporating both continuing infectious disease burdens and the developing burden of NCDs through effective intersectoral cooperation.

## Conclusions

Similar to many low-income Asian countries, performance in achieving the health-related MDGs has not been uniform in Myanmar. Countries like Cambodia and Bangladesh, which have already achieved or are on track to achieve MDG 6 targets and with socio-economic features roughly comparable to Myanmar’s, have made significant reductions in child and maternal mortality over the past 15 years [30,31], while Myanmar still needs to step up progress in these areas. Cambodia significantly increased institutional delivery and skilled birth attendance rates as well as eliminating financial barriers to maternity care for low-income women by voucher schemes [31]. Bangladesh, meanwhile, has brought about significant improvements in vitamin A supplementation and control of diarrheal diseases to reduce child and maternal mortality [30]. Such experiences may prove instructive in Myanmar’s own efforts to improve child and maternal mortality.

Though Myanmar has made demonstrable progress on health over the past two decades (especially regarding MDG 6) much remains to be done beyond 2015 to sustain the gains that have been made to date and to ensure more equitable levels of achievement across populations and programs. Moreover, the post-2015 development agenda must reflect a broader array of “new” issues includes NCDs, health systems strengthening, and universal health coverage. Leading up to the 2015 MDGs cut-off and beyond, developing countries like Myanmar must focus on cultivating strong, effective, and efficient health systems for provision of quality services. This requires sufficient commitment and funds for the health sector, basic infrastructure, human resources, and a solid health systems and data management structure.

## Abbreviations

ART: Antiretroviral therapy; CTHP: Coordinated township health plan; DOTS: Directly observed treatment, short-course; EPI: Expanded programme on immunization; FHAM: Fund for HIV/AIDS in Myanmar; GAVI: Global alliance for vaccines and immunizations; GDP: gross domestic product; HDI: Human development index; HSS: Health system strengthening; IMR: Infant mortality rate; ITN: Insecticide-treated net; LLIN: Long-lasting insecticide-treated net; MDGs: Millennium Development Goals; MMR: Maternal mortality rate; MOH: Ministry of Health, Myanmar; NCDs: Non-communicable diseases; NGO: Non-governmental organization; NHC: National health committee; NHP: National health plan; NSP: National strategic plan on HIV and AIDS; ODA: Official development assistance; OOP: Out-of-pocket (expense); PMTCT: Prevention of mother-to-child transmission; RHCs: Rural health centers; TB: Tuberculosis; U5MR: Under-five mortality rate; UNAIDS: Joint United Nations Programme on HIV/AIDS.

## Competing interests

The authors declare that they have no competing interests.

## Authors’ contributions

YMS, KLW, LWS, MMT and ST conducted the literature search, reviewed articles, synthesized findings, and drafted the manuscript. RMA and AS contributed to the synthesis and provided substantive inputs and edits on drafts of the manuscript. MJ provided guidance on the framework and direction of the literature review, along with critical input on manuscript drafts. All authors reviewed, revised, and approved the final manuscript.
